# A Proterozoic microbial origin of extant cyanide-hydrolyzing enzyme diversity

**DOI:** 10.3389/fmicb.2023.1130310

**Published:** 2023-03-30

**Authors:** Sarah L. Schwartz, L. Thiberio Rangel, Jack G. Payette, Gregory P. Fournier

**Affiliations:** ^1^Department of Civil and Environmental Engineering, University of California, Berkeley, Berkeley, CA, United States; ^2^Graduate Program in Microbiology, Massachusetts Institute of Technology, Cambridge, MA, United States; ^3^Department of Earth, Atmospheric and Planetary Sciences, Massachusetts Institute of Technology, Cambridge, MA, United States

**Keywords:** nitrile, nitrilase, cyanide, nitrile hydratase, thiocyanate hydrolase, molecular clock, phylogenetics

## Abstract

In addition to its role as a toxic environmental contaminant, cyanide has been hypothesized to play a key role in prebiotic chemistry and early biogeochemical evolution. While cyanide-hydrolyzing enzymes have been studied and engineered for bioremediation, the extant diversity of these enzymes remains underexplored. Additionally, the age and evolution of microbial cyanide metabolisms is poorly constrained. Here we provide comprehensive phylogenetic and molecular clock analyses of the distribution and evolution of the Class I nitrilases, thiocyanate hydrolases, and nitrile hydratases. Molecular clock analyses indicate that bacterial cyanide-reducing nitrilases were present by the Paleo- to Mesoproterozoic, and were subsequently horizontally transferred into eukaryotes. These results present a broad diversity of microbial enzymes that could be optimized for cyanide bioremediation.

## Introduction

Cyanide is well-known as a globally distributed environmental pollutant. The anion (CN^−^), which is produced as a byproduct in several industries, including mining, electroplating, petrochemical refining, and pharmaceutical manufacturing, poses a significant health hazard (Raybuck, 1992; Ebbs, 2004; Alvillo-Rivera et al., 2021). Free CN^−^ anions and hydrogen cyanide (HCN) inhibit aerobic respiration by binding metalloproteins such as cytochrome *c* oxidase, and are extremely toxic to animals at high concentrations (Raybuck, 1992). Additionally, HCN can disrupt biogeochemical cycling, especially within the nitrogen cycle (Kapoor et al., 2016). However, cyanide is also produced biologically, and diverse plants, fungi, and bacteria have well-established enzymatic pathways for free cyanide metabolism (Raybuck, 1992; Ebbs, 2004; Benedik and Sewell, 2017; Park et al., 2017). As a result, these organisms have been studied and engineered for bioremediation applications in cyanide-contaminated areas (Martínková et al., 2015; Park et al., 2017).

**Figure 1:**
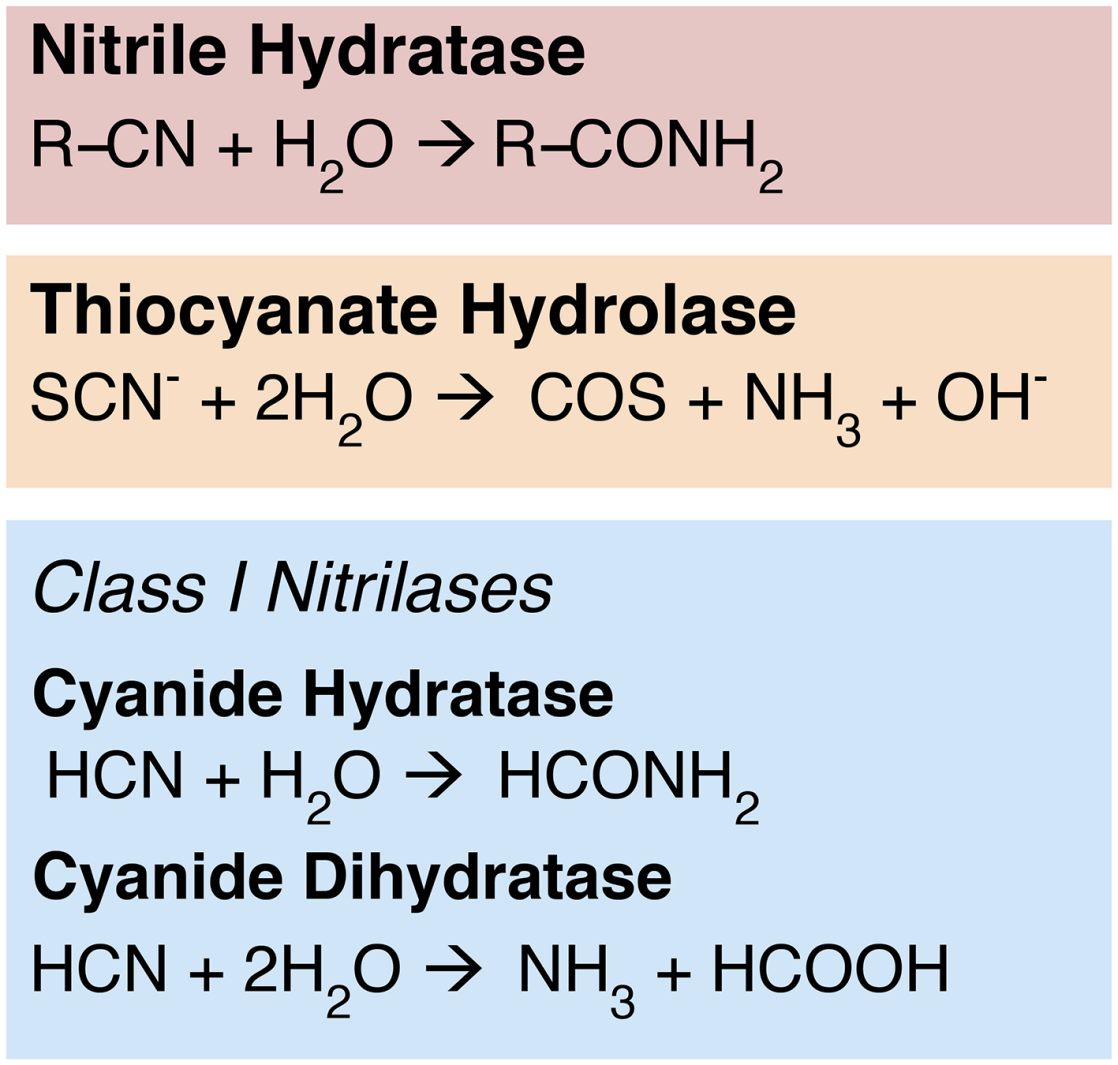
Nitrile-hydrolyzing enzymes. Nitrilases are nonhomologous to NHases and SCNases, but display some convergent function: While cyanide dihydratase exhibits classical nitrilase activity—producing ammonia and a carboxylic acid—cyanide hydratase exhibits NHase-like chemistry, producing an amide product.

Multiple enzyme families have the capacity to reduce the strong nitrile bond between carbon and nitrogen. For example, the nitrile hydratases (NHases) convert organic nitrile substrates to corresponding amide products (Figure 1), a property that has been utilized in industrial applications such as the production of acrylamide or nicotinamide (Kobayashi and Shimizu, 1998, 2000; Cheng et al., 2020). These two-subunit enzymes require iron or cobalt cofactors; cobalt NHases are further characterized into either high or low molecular weight groups (Kobayashi and Shimizu, 2000). The homologous enzyme thiocyanate hydrolase (SCNase) reduces the nitrile bond in thiocyanate (Figure 1; Kobayashi and Shimizu, 2000; Ebbs, 2004); these enzymes are central players in the bioremediation of thiocyanate-contaminated mining and industrial sites (Kobayashi and Shimizu, 2000; Kantor et al., 2015).

While functionally similar to SCNase and NHase, enzymes within the Class 1 nitrilase subfamily exhibit more targeted substrate specificity toward free HCN. The Class 1 nitrilases are members of the greater nitrilase superfamily, which contains over a dozen other subfamilies with varying distributions and substrate specificities (Pace and Brenner, 2001). The physiological role of nitrilases has not been entirely elucidated, but the superfamily encompasses diverse functionality ranging from lipoprotein modification to nucleic acid catabolism and protein deamination (Pace and Brenner, 2001; Howden and Preston, 2009; Thuku et al., 2009; Chhiba-Govindjee et al., 2019). Mammalian nitrilase homologs are associated with cell growth, vitamin synthesis, and tumor suppression (Pace and Brenner, 2001; Barglow et al., 2008; Zhang et al., 2009; Silva Teixeira et al., 2021). Plant nitrilases play a role in hormone biosynthesis, nitrogen recycling, and detoxification of nitrile-containing compounds and intermediates (Bestwick et al., 1993; Kobayashi et al., 1993; Kato et al., 2000; Piotrowski, 2008; Janowitz et al., 2009). Fungal and bacterial nitrilases have been hypothesized to play a similar detoxification role for nitrile-containing compounds, and may also be involved in biosynthesis of secondary metabolites (Podar et al., 2005; Howden and Preston, 2009; Martínková et al., 2009).

Class 1 nitrilases in particular are found in diverse bacteria, fungi, and plants (Pace and Brenner, 2001). The cyanide-degrading Class 1 nitrilases include both cyanide hydratase (CHT), which is primarily found in fungi, and cyanide dihydratase (CynD), which is thought to be primarily bacterial in distribution (Ebbs, 2004; Benedik and Sewell, 2017). Although CHT and CynD are closely related, with catalytic sites that are currently indistinguishable in primary sequence, their biochemistry differs slightly (Figure 1).

CynD exhibits the canonical “nitrilase” activity characteristic of the broader superfamily, reducing a nitrile-containing substrate to the corresponding carboxylic acid and ammonium (Podar et al., 2005; Martínková et al., 2015; Benedik and Sewell, 2017). CHT, however, performs the same biochemistry as NHase, cleaving and converting a nitrile group to an amide (formamide); it is believed that this amide product may be subsequently reduced further *in situ* by another nitrilase superfamily enzyme, amidase (Ebbs, 2004; Benedik and Sewell, 2017). Like NHases, nitrilases have been explored for industrial or bioremediation applications (Pace and Brenner, 2001; DeSantis et al., 2002; Gong et al., 2012; Benedik and Sewell, 2017; Chhiba-Govindjee et al., 2019). Despite the functional similarities of CHT and NHase, nitrilases and NHases have low sequence similarity and very likely do not have shared ancestry (O’Reilly and Turner, 2003). Genomic context for Class 1 nitrilases is not conserved between major groups, and these genes do not appear to be transcribed together with other nitrogen metabolism genes (Supplementary Figure S1). This is consistent with an evolutionary history marked by horizontal gene transfer, which generally disrupts gene synteny between groups.

Class 1 nitrilases provide a diverse array of enzymes that could be harnessed for cyanide bioremediation or industrial applications. However, this subfamily may also enable exploration of questions about Earth history and early microbial life. CHT and CynD have broad taxonomic distributions and do not require molecular oxygen for catalysis, suggesting they could be relatively ancient enzymes. Therefore, nitrilases are useful targets for molecular clock dating to constrain the age of microbial cyanide metabolism. Such analyses provide independent biological context to constrain the presence of HCN on early Earth. Beyond broadening our understanding of the evolution of the planet’s nitrogen cycle, this context can also help constrain hypotheses suggesting a central role for HCN in early biochemistry.

HCN has long been viewed as a leading candidate among hypothesized feedstock molecules for abiogenesis. Studies indicate that various abiotic processes, including photochemistry and oligomerization, can transform HCN into a wide variety of nucleic and amino acids including adenine, alanine, glycine, and aspartic acid (Oró, 1961; Oró and Kamat, 1961; Abelson, 1966; Ferris et al., 1978; Draganić et al., 1980). Models suggest that this chemistry could have been plausibly supported by atmospheric conditions and a small but sufficient standing stock of HCN on the early Earth (Zahnle, 1986; Sutherland, 2016). Archean HCN concentrations are expected to have increased further following the emergence of methanogens, as increased levels of atmospheric methane drive deposition of HCN (Tian et al., 2011). If HCN was indeed a key prebiotic feedstock, and increasingly abundant into the Archean eon, this would have provided a key substrate for early microbial enzymes, which may be ancestral to extant enzymatic families.

Reconstructing the evolutionary history of modern HCN-metabolizing enzymes therefore provides a novel approach for constraining the presence and nature of early biochemical systems. Although over six decades of chemical syntheses and planetary modeling have produced extensive data supporting HCN’s hypothesized role in the origin or early evolution of life, few genomic investigations have attempted to inform or constrain these models. Some hypotheses have suggested that cyanide biochemistry by nitrilases could in fact be quite young, with HCN-hydrolyzing nitrilases evolving in stem eukaryotes and later undergoing horizontal transfer from plants or animals to bacteria and archaea (Pace and Brenner, 2001). This scenario would suggest that nitrilases were not selected for until relatively recently in Earth’s history, inconsistent with hypotheses that cyanide was an abundant, important substrate for microbial life before this. However, if nitrilases emerged in older prokaryotic lineages, this would suggest a much earlier presence and significance for HCN in nitrogen biogeochemistry.

## Materials and methods

### Sequence sampling and HMM construction

#### Nitrilases

Representative amino acid sequences were selected for cyanide dihydratase (*Bacillus pumilus*, GenBank AAN77004.1) and cyanide hydratase (*Neurospora crassa*, GenBank XP_960160.2; Benedik and Sewell, 2017). Each representative sequence was used as a query to search the conservative UniRef90 protein database (The UniProt Consortium, 2021) *via* NCBI’s Basic Local Alignment Search Tool (BLAST; Camacho et al., 2009). Candidate homolog sequences were obtained from reported hits by only including sequences with query sequence identity ≥70%; query sequence coverage ≥95%; and E-value <10^−10^. Only sequences of 200–500 residues in length were included to eliminate alignment artifacts arising from partial or multienzyme sequences. Filtered hits were aligned using Muscle (Edgar, 2004) and used to construct a Hidden Markov Model (HMM) profile using hmmbuild in the HMMer package (Eddy, 2011). The HMMer profile was used to search NCBI’s non-redundant protein database (Agarwala et al., 2018) as of January 2019 for more distant homolog candidates. Resulting hits were filtered for quality; partial hits and hits with e-value >10^−10^ were removed. Only sequences with length 50–500 residues in length were included. Resulting hits were subsampled to a single representative per genus to control for variance in database representation between genera. Candidate homologs reported by hmmsearch were aligned using Muscle (Edgar, 2004). The resulting alignment was analyzed, and the conserved catalytic triad for nitrilases (Pace and Brenner, 2001) was identified by residue analysis at sites Glu-578, Lys-988, and Cys-1205 (alignment data available in https://doi.org/10.6084/m9.figshare.c.5952186). Sequences with deletions at or adjacent to the conserved catalytic sites (29 sequences total) were removed, to improve the likelihood of retaining true nitrilase homologs. Isolated sequences with apparent autapomorphic insertions of ≥20 residues (10 sequences total) were also removed to avoid alignment artifacts. The remaining 1,232 sequences were realigned using Muscle (Edgar, 2004).

To reduce site-saturation and simplify downstream tree topology for visualization, the initial alignment was further subsampled; the outgroup (identified as primarily Class 10/Class 13 nitrilases *via* conserved domain analysis based on an exploratory tree) was subsampled to one member from each clade with class-level monophyly; two members of each clade with class-level monophyly were retained in Metazoa for downstream molecular clock calibrations. The ingroup (identified as primarily Class 1 nitrilases *via* conserved domain analysis in an initial tree) was subsampled to two members of each clade with order-level monophyly. Two sequences with putative assembly or annotation errors (XP_019577256.1 and XP_005975261.1) were removed from the dataset, and remaining sequences were realigned with MAFFT (Nakamura et al., 2018), using automatic parameterization. The alignment was manually edited to exclude all sites before site 286 and after site 1,456, as these N- and C-terminal regions were gap-rich in comparison to the rest of the alignment. The resulting alignment was used for gene tree construction and visualization.

For molecular clock analyses, the alignment used for tree construction was further subsampled to one representative per clade with phylum-level monophyly, except for groups containing taxa with available fossil calibrations (see below). Sequence XP_023622267.1 was removed due to the presence of ambiguous sequence characters that prevented conserved motif characterization. Each taxon was assigned a unique numerical identifier (a table of numerical identifiers with corresponding taxon names and NCBI accession numbers in the repository, https://doi.org/10.6084/m9.figshare.c.5952186). This alignment was used to construct a maximum-likelihood tree in IQ-Tree, using the optimal substitution model identified under the Bayes Information Criterion (BIC): (LG+F+R7). This tree was used as the starting topology for molecular clock analyses (see below).

Gene neighborhood analyses for nitrilase homologs were performed for a 10 kb region around selected nitrilase homologs using Gene Graphics (Harrison et al., 2018).

#### Nitrile hydratases (NHases)

Three representative sequences were used for the NHase beta subunit: one iron-type enzyme from *Psuedomonas chlororaphis* (Nishiyama et al., 1991; GenBank BAA14246.1), one low-molecular weight cobalt-type enzyme from *Rhodococcus rhodocrous* (Kobayashi et al., 1991) (GenBank CAA45711.1), and one high-molecular weight cobalt-type enzyme from *Rhodococcus rhodocrous* (Kobayashi et al., 1991; GenBank CAA45709.1). Each sequence was used to independently BLAST search the UniRef90 database. Resulting hits were filtered to retain only hits with e-value <10^−10^, sequence identity ≥50%, and query coverage ≥75%. The filtered hits were combined and aligned in Muscle. This alignment was used for HMM construction; the resulting HMM was used to search NCBI’s non-redundant protein database. Resulting hits were filtered to exclude hits with e-value >10^−10^ and partial sequences. Sequence length of hits was plotted with a Seaborn KDE plot, and sequences <200 and > 300 residues in length were excluded from the dataset. The remaining sequences were aligned with Muscle (Edgar, 2004). The alignment was manually checked, and four likely misaligned sequences were removed from the dataset (WP_012455950.1, WP_043079656.1, WP_085127903.1, WP_013808494.1). The remaining sequences were realigned using Muscle (Edgar, 2004), and the resulting alignment used for ML tree construction.

#### Thiocyanate hydrolases (SCNases)

A representative thiocyanate hydrolase from *Thiobacillus thioparus* (NCBI reference sequence WP_018507189.1) was used as a query to BLAST the conservative UniRef90 protein database. Resulting hits were filtered to retain only hits with e-value <10^−10^, sequence identity ≥50%, and query coverage ≥75%. The resulting hits were aligned in Muscle (Edgar, 2004), and the alignment was used to construct an HMM using HMMer. The HMM was used to search NCBI’s non-redundant protein database. Resulting hits were filtered to exclude hits with e-value >10^−10^ and partial sequences. Sequence lengths of hits were plotted using a Seaborn KDE plot, and hits <175 or > 300 residues in length were removed from the dataset. Resulting hits were subsampled to one representative per genus. Two sequences with significant missing data (WP_089128740.1, PYJ67070.1) were removed from the dataset. The remaining sequences were re-aligned with Muscle (Edgar, 2004); the resulting alignment was used for maximum-likelihood (ML) tree construction. An initial tree revealed an anomalous long branch for one taxon (*Methylobateraceae* WP_081435408.1); to avoid possible long branch attraction artifacts, this taxon was removed, the remaining taxa realigned with MAFFT with automatic model selection, and this alignment used for ML tree construction.

### Tree construction

#### Nitrilases

**Figure 2:**
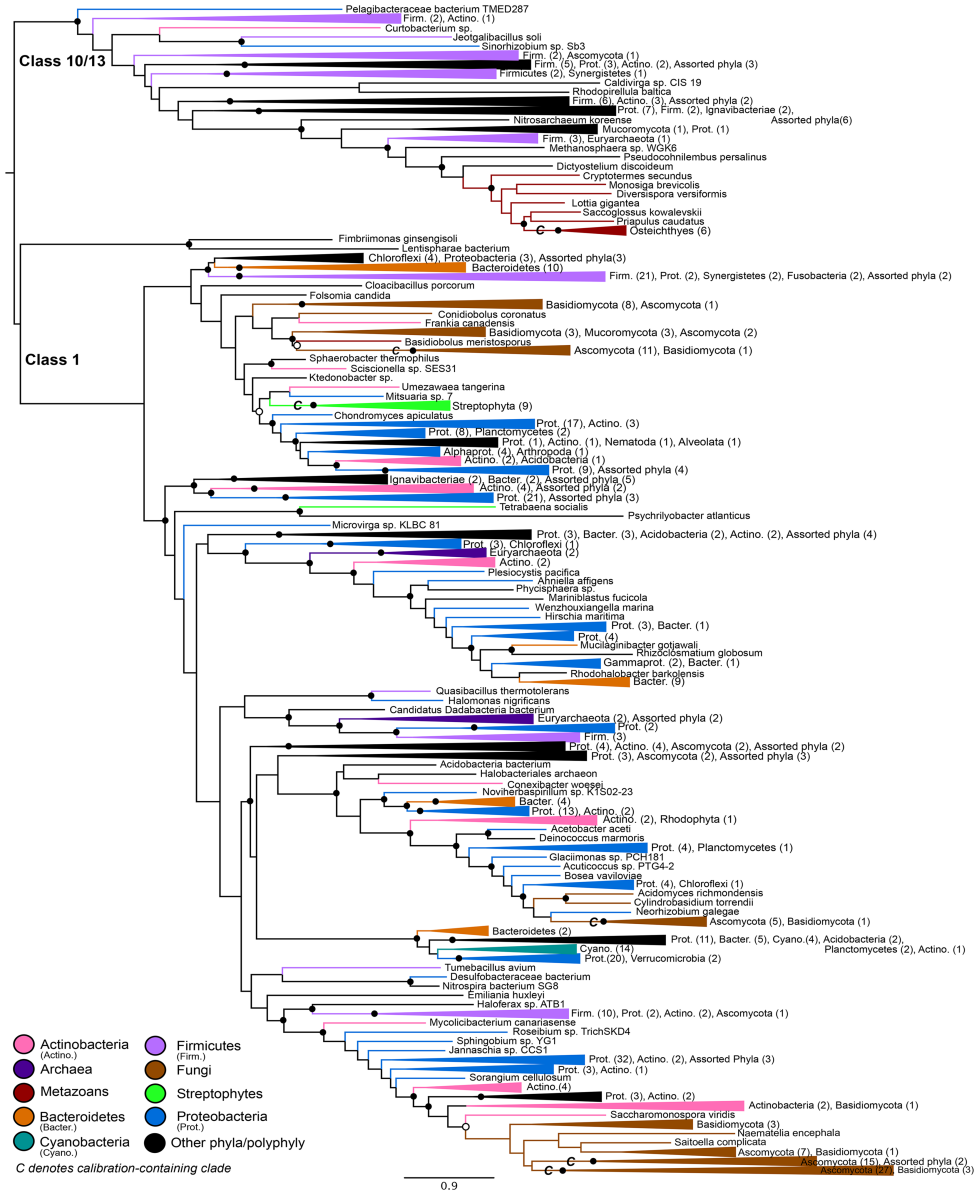
Nitrilase gene tree. Clades containing over 67% representation of a single group are color-coded as shown; taxon counts and sub-phylum level taxonomies are labeled on the tree. Isolated representatives of given phyla (due to subsampling for visualization) are described as “assorted phyla” within paraphyletic clades. Node supports are colored by the values of both approximate likelihood ratio test (aLRT) and rapid bootstrap (UFBoot) values: Strong support with both aLRT/UFBoot values ≥90 (black), weak support with both values ≤50 (white). Unlabeled nodes have either intermediate aLRT/bb support (one or both values between 50 and 90) or conflicting support (one value ≥90 and the other ≤50).

An initial maximum likelihood gene tree (available in https://doi.org/10.6084/m9.figshare.c.5952186) was constructed in IQ-Tree (Nguyen et al., 2015) using the optimal substitution model based on BIC: LG+I+G4. A subsampled alignment (see above) was used to construct a simplified maximum-likelihood gene tree (Figure 2) in IQ-Tree using the optimal substitution model: LG+F+R10. Statistical supports for bipartitions were evaluated using IQ-Tree’s approximate-likelihood ratio test (aLRT) and UltraFast Bootstrap (UFBoot) metrics. The resulting gene tree was rooted using Minimal Ancestor Deviation (MAD) rooting (Tria et al., 2017). This rooting was determined to be congruent with a manual rooting between the Class 1 nitrilases and the Class 10/Class 13 nitrilases, as determined by conserved domain analysis (Marchler-Bauer et al., 2015; Lu et al., 2020).

#### NHases and SCNases

The NHase alignment and SCNase alignment were used to construct maximum-likelihood gene trees using IQ-Tree, using the optimal substitution models identified under the BIC: LG+F+R7 (NHase) and WAG+F+R9 (SCNase). Statistical supports for bipartitions were evaluated using IQ-Tree’s approximate-likelihood ratio test (aLRT) and UltraFast Bootstrap (UFBoot) metrics. The resulting gene trees were rooted using MAD.

## Molecular clock analyses

### Constraint optimization

Initial molecular clocks were constructed in Phylobayes v. 4.1c (Si Quang et al., 2008) under a normally-distributed root prior of 3.8–1.0 Ga to evaluate possible diversification ages from the Archean through the mid-Proterozoic. Initial plant, fungal, and animal secondary fossil calibrations were selected from the literature based upon representation of groups in the nitrilase gene tree; this included an initial animal calibration constraining crown Bilateria with an age of 688–596 Ma (Dos Reis et al., 2015), calibrating the LCA of *Cryptotermes secundus* (tax439) and *Clupea harengus* (tax467) in the tree (Supplementary Figure S2). Analysis of the initial clocks revealed minimum predicted root ages just over 1.0 Ga; as a result, subsequent runs relaxed the minimum age on the root prior to 800 Ma, to ensure that posterior root age estimates were not artificially constrained nor overdetermined by the selected prior. This initial animal calibration consistently failed to predict accurate age ranges of clades with known divergence times, resulting in unrealistically ancient uncalibrated age estimates for plant and fungal linages in the tree. Because the Bilateria were under-sampled in some basal groups (Supplementary Figure S2), a more conservative crown Osteichthyes calibration was used for subsequent analyses (see below).

### Calibrations

Molecular clocks were run under a normally-distributed root prior of 3.8–0.8 Ga. In addition to the root prior, prior and posterior distributions were calculated with the following additional sets of secondary fossil constraints:

1. No additional calibrations (root prior only).
2. Plant calibration: The Charophyta-Klebsormidiophyta/land plant clade was constrained with an age of 750–590 Ma (Morris et al., 2018), calibrating the last common ancestor (LCA) of *Brassica oleracea* (tax247) and *Chara braunii* (tax314) in the tree.
3. Animal calibration: The Osteichthyes (Euteleostomi) were constrained with an age of 444–421 Ma (Dos Reis et al., 2015), calibrating the LCA of *Pelodiscus sinensis* (tax445) and *Clupea harengus* (tax467).
4. Fungal calibrations:
  - Three clades in the tree represented the Sordariomycetes/Dothideomycetes/Leotiomycetes clade, all constrained with an age of 350–250 Ma (Prieto and Wedin, 2013), calibrating the LCAs of *Sporothrix insectorum* (tax222) and *Fibularhizoctonia* sp. *CBS 109695* (tax331); *Verruconis gallopava* (tax51) and *Amorphotheca resinae* (tax82); and *Cenococcum* (tax24) and *Pseudomassariella* (tax 4).
  - The Pezizomycetes/Leotiomycetes clade was constrained with an age of 490–400 Ma (Prieto and Wedin, 2013), calibrating the LCA of *Morchella* (tax380) and *Ustilaginoidea* (tax384).
  - The crown Ascomycota were constrained with an age of 680–410 Ma (Prieto and Wedin, 2013), calibrating the LCA of *Hyaloscypha variabilis* (tax61) and *Pyronema omphalodes CBS 100304* (tax87).
5. Plant and animal calibrations (sets 2 and 3).
6. Plant and fungal calibrations (sets 2 and 4).
7. Animal and fungal calibrations (sets 3 and 4).
8. All (Plant, animal, and fungal) calibrations (sets 2, 3, and 4).

To assess the relative consistency of clock models, variance and population standard deviations were calculated for normally distributed age estimates between the three clades constrained by the same Sodariomycetes/Dothideomycetes/Leotiomycetes calibration (4a above).

### Models and parameters

To evaluate the effect and limit the bias of model selection on age estimates, clocks for all calibration subsets were run under three different clock models: uncorrelated gamma multipliers (Drummond et al., 2006; UGAM), autocorrelated lognormal (Thorne et al., 1998) (LN) and the autocorrelated CIR model (Lepage et al., 2006; CIR). Clocks were run under a fixed C20 empirical profile mixture model (Si Quang et al., 2008). Clocks were run with two chains, and convergence was assessed as effective size ≥50 and variable discrepancies ≤0.30 for all parameters.

## Results

### Nitrilases

The maximum-likelihood gene tree for nitrilases includes diverse representatives from all three domains of life (Figure 2). Conserved domain analysis indicates that the outgroup to the Class 1 nitrilases represented in the tree comprises primarily members of the Class 10 and Class 13 nitrilases. This outgroup contains a monophyletic group of metazoans; at the class level, these taxa roughly follow predicted species tree topology for representatives of the Osteichthyes (Euteleostei) and shallower crown bilaterians (Supplementary Figure S2).

The Class 1 nitrilases are particularly enriched in occurrence within bacteria and fungi, consistent with previously described phylogenetic distributions of cyanide dihydratase and cyanide hydratase. Proteobacteria, Ascomycota, and Basidiomycota are particularly highly represented compared with other phyla. Several groups in the nitrilase gene tree show phylum- or class-level monophyly, including a clade of plants that is consistent with the species tree topology for Streptophyta (Supplementary Figure S3; Morris et al., 2018). Conserved domain analysis indicates that this clade contains the Nit6803 nitrilases. Such monophyly indicates a deep history of vertical inheritance of Class 1 nitrilases. However, multiple instances of polyphyly and inconsistency with prokaryotic and eukaryotic species tree topologies additionally indicates several horizontal gene transfers. Notably, eukaryotic groups do not place together within the tree, but instead are independently nested within larger bacterial groups. This topology is consistent with multiple independent horizontal transfers of nitrilase genes from different bacterial lineages into eukaryotic groups. The limited taxonomic diversity within various microbial lineages, and the relative closeness of these lineages to eukaryal groups in the tree, further argues for a history of extensive HGT, rather than a species tree topology obscured by poor phylogenetic signal or tree reconstruction artifacts.

**Figure 3:**
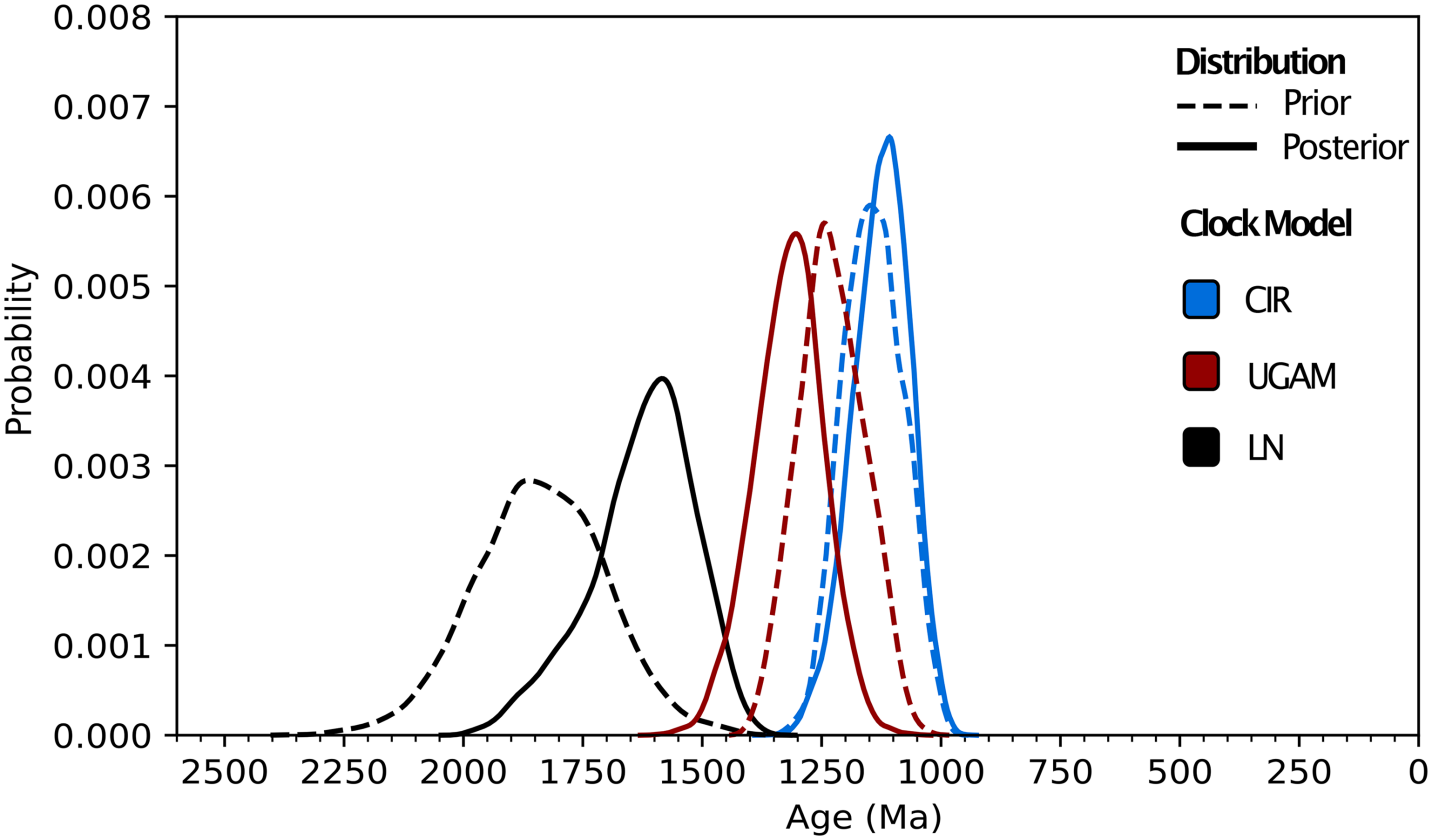
Molecular clock distributions. Prior and posterior age distributions for the last common ancestor of Class 1 nitrilases are shown for uncorrelated gamma (UGAM), lognormal (LN), and CIR process clock models. All distributions shown are run using all animal, plant, and fungal fossil calibrations (see “Methods”) and a permissive root prior of 3,800–800 Ma.

Molecular clock analyses including all calibrations predict Class 1 nitrilases having a recent common ancestor between 1.88 and 1.02 Ga (Figure 3; Supplementary Table S1). The estimated range and mean age for the Class 1 ancestor varies by clock model: LN predicts the oldest age range and mean age (1.88–1.45 Ga and 1.63 Ga, respectively), CIR predicts the youngest age range and mean age (1.02–1.25 Ga and 1.13 Ga, respectively), and UGAM offers intermediate estimates for age range and mean age (1.57–1.18 Ga and 1.32 Ga, respectively). The calibration-specific analyses also indicate that the plant calibration is most-informative for recovering the fossil-constrained ages of the other calibrated clades, while the animal calibration often overestimates—and the fungal calibration routinely underestimates—these constrained ages (Supplementary Table S2).

**Figure 4:**
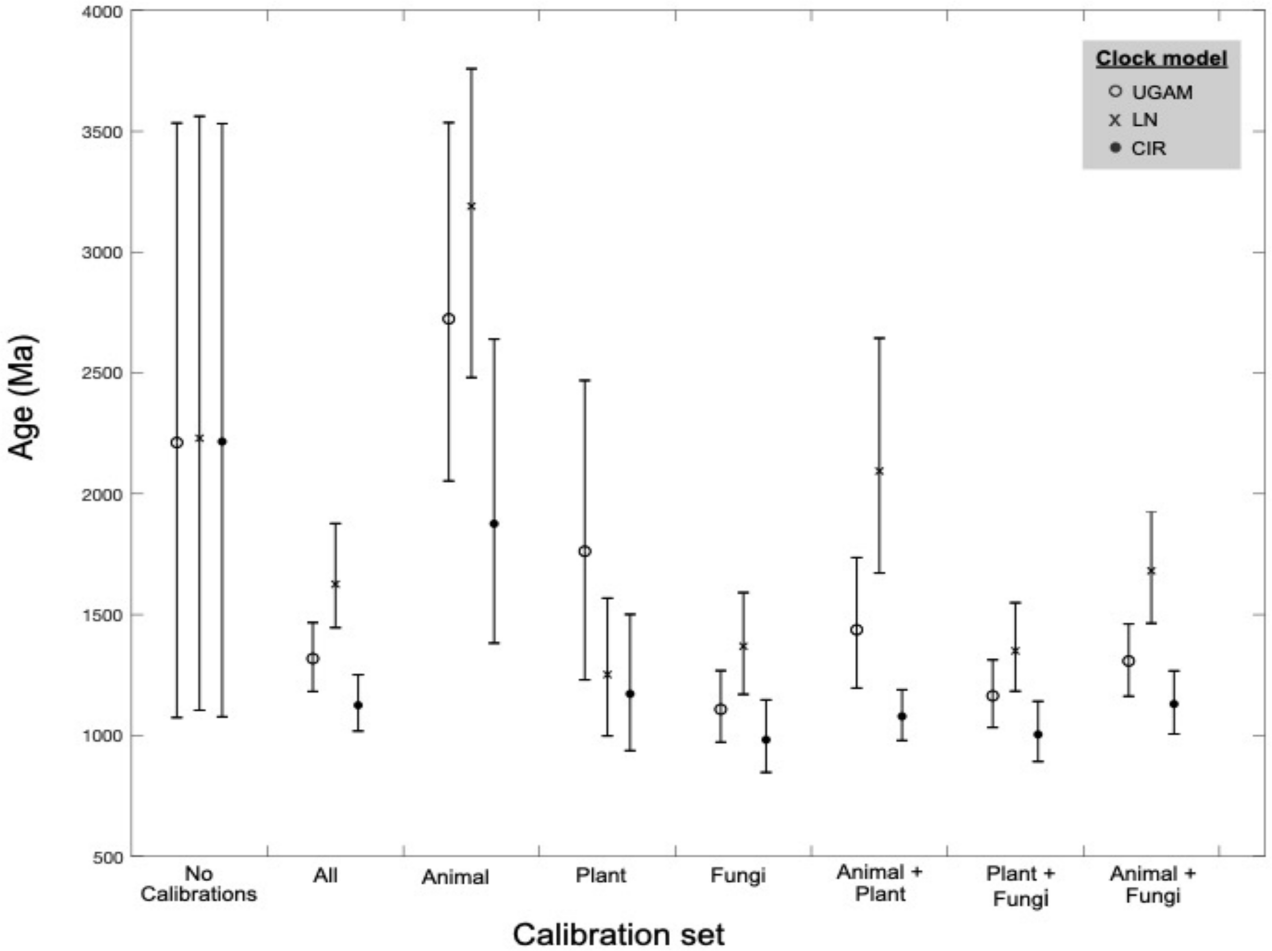
Molecular clock age estimates by model and calibration set. Mean age (markers) and age range (bars) estimates are shown for molecular clock posterior distributions by calibration subset. Three clock models were tested for each calibration subset (see marker type).

Across different combinations and subsets of fossil calibrations, UGAM and CIR clock models offer age estimates that are more consistent with one another than the LN model (Figure 4), suggesting that UGAM and CIR may provide more reliable dates. This result is further supported through variance analyses between the three fungal clades with the same calibration. The LN model fails to minimize the variance for any relevant calibration subset, for prior or posterior distributions (Supplementary Table S3); this is consistent with the apparent calibration sensitivity and variability observed for LN in the calibration-specific analyses. Together, these data suggest that LN may provide the least-reliable age estimates of the three tested clock models—and CIR may provide the most-reliable estimates. These analyses also underscore the utility of duplicate gene transfers into calibration-constrained clades as a tool for adding rigor in clock model optimization.

### Nitrile hydratases and thiocyanate hydrolases

**Figure 5:**
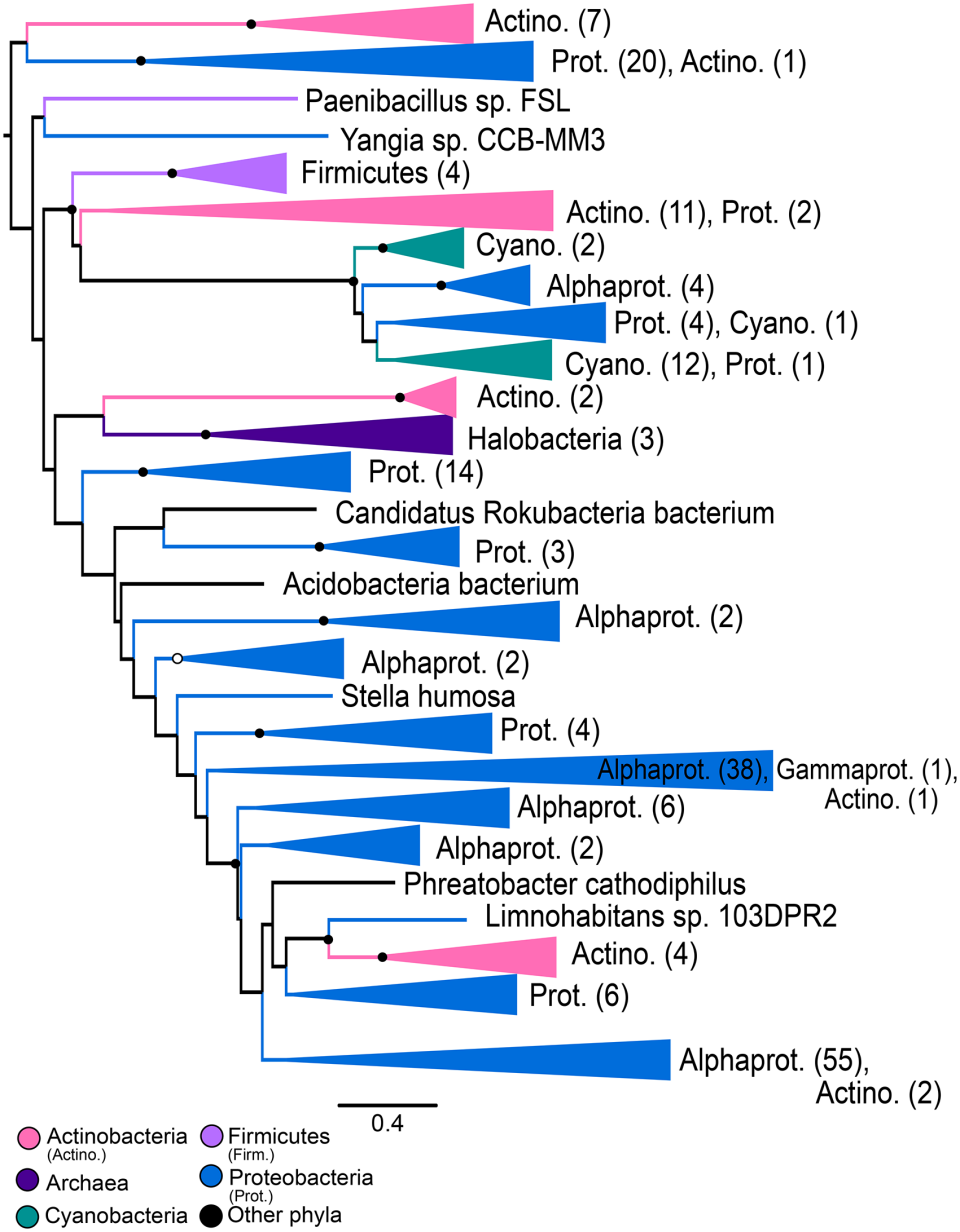
Nitrile hydratase gene tree. Clades containing over 67% representation of a single group are color-coded as shown; taxon counts and sub-phylum level taxonomies are labeled on the tree. Node supports are colored by the values of both approximate likelihood ratio test (aLRT) and rapid bootstrap (UFBoot) values: Strong support with both aLRT/UFBoot values ≥90 (black), weak support with both values ≤50 (white). Unlabeled nodes have either intermediate aLRT/UFBoot support (one or both values between 50 and 90) or conflicting support (one value ≥90 and the other ≤50).

**Figure 6:**
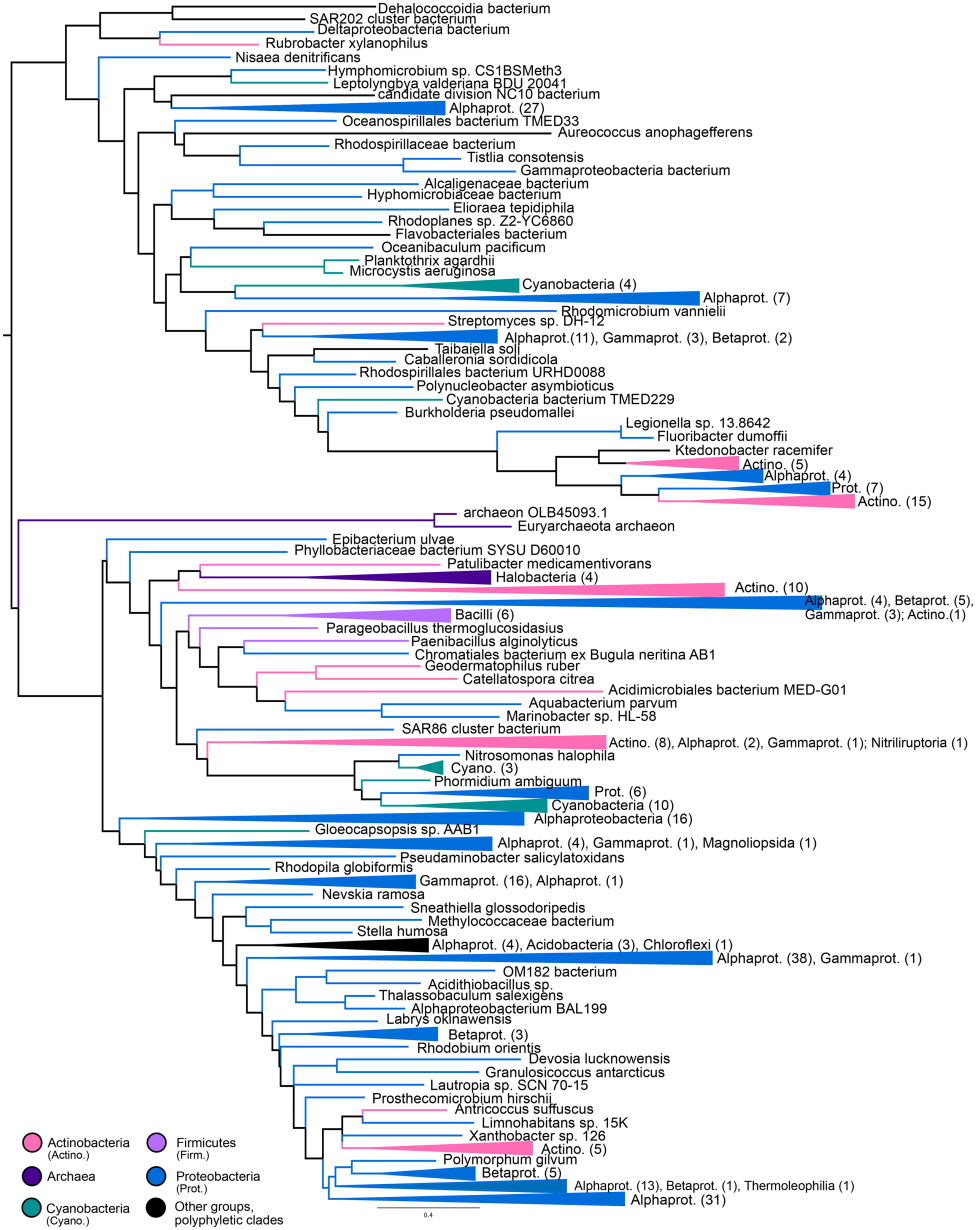
Thiocyanate hydrolase gene tree. Clades containing over 67% representation of a single group are color-coded as shown; taxon counts and sub-phylum level taxonomies are labeled on the tree. Node supports are colored by the values of both approximate likelihood ratio test (aLRT) and rapid bootstrap (UFBoot) values: Strong support with both aLRT/UFBoot values ≥90 (black), weak support with both values ≤50 (white). Unlabeled nodes have either intermediate aLRT/UFBoot support (one or both values between 50 and 90) or conflicting support (one value ≥90 and the other ≤50).

Gene trees for NHases (Figure 5) and SCNases (Figure 6) reveal similar taxonomic distributions for these homologous enzymes. Both enzymes appear exclusively prokaryotic in distribution. Most orthologs are within members of the Proteobacteria, with particular enrichment among Alphaproteobacteria; Actinobacteria are also well-represented. Both NHases and SCNases are also found in members of the Cyanobacteria, Firmicutes (specifically Bacilli), and halophilic Archaea. Though both trees contain multiple groups with phylum-level monophyly (including within Bacilli and Archaea), the trees also show substantial polyphyly, especially for Proteobacteria, which are represented at all depths of the tree. This limited sampling of monophyletic groups and an overall paucity of well-established fossil representatives suggest that the NHase and SCNase trees are poor candidates for molecular dating.

## Discussion

The topology of the nitrilase tree does not support hypotheses proposing that the nitrilase ancestor originated in eukaryotes and was subsequently transferred into bacteria. It is not possible to completely rule out multiple lineage-specific losses of an ancestral eukaryotic nitrilase gene; however, the phylogenetic distance observed between eukaryotic groups, the nested placement of eukaryotic groups within bacterial-enriched clades, and the taxonomic monophyly observed within sampled plants and animals all support a bacterial origin with multiple subsequent horizontal transfers into eukaryotic groups.

Molecular clock dating also supports a prokaryotic origin for the nitrilase ancestor. The CIR clock model predicts the youngest Class 1 nitrilase ancestor age (1.13 Ga mean age, with a minimum age range extending to 1.02 Ga) among models run with all fossil calibrations (Figures 3, 4; Supplementary Table S1). While some of the older age range estimates predicted by other clock models and calibration subsets in this work do, in fact, exceed older-bound age estimates for the last eukaryotic common ancestor at ~1.9 Ga (Penny et al., 2014), this youngest Proterozoic age estimate does not itself preclude a nitrilase origin in ancestral eukaryotes. However, existing hypotheses suggest that ancestral nitrilases diversified in animals and plants *before* horizontal transfer into microbial lineages (Pace and Brenner, 2001). The last common ancestors of crown Viridiplantae, crown Metazoa, and crown Ascomycota are predicted to be, at maximum, 972 Ma (Morris et al., 2018), 834 Ma (Dos Reis et al., 2015), and 671 Ma (Prieto and Wedin, 2013), respectively—all younger than the lowest bound of the youngest predicted age distribution for nitrilases. The predicted age of the nitrilases in the tree is therefore inconsistent with the diversification of these enzymes within eukaryotes. Together with the gene tree topologies, these results support a prokaryotic, Proterozoic origin for extant HCN-hydrolyzing nitrilase protein families.

It is not clear what sources of HCN were present during the Paleo- to Mesoproterozoic, as this period postdates proposed prebiotic synthesis processes in operation during the Archean, yet precedes the evolution of cyanogenic plants. Therefore, this work intriguingly implies that another microbiogenic or abiotic source of free cyanide was generally available during the Proterozoic. Since this molecular clock data recovers a younger-bound estimate for this ancestral age, and lineage-specific losses or other coalescent processes may push convergence times younger, the true ancestry of this gene family may well extend into the Archean. Nevertheless, in order for these enzyme families to have persisted, HCN must have been available continually during the intervening Proterozoic Eon.

While these results support an earlier origin for nitrilases than previously proposed, these specific nitrilase enzyme families may not represent the earliest form of microbial cyanide metabolism. Other nitrile-hydrolyzing enzymes such as NHase or SCNase could predate nitrilases, but are difficult to temporally constrain due to their limited taxonomic distribution and extensive transfer history. Other extinct enzyme families, or extant enzyme families that are not adapted for HCN or nitrile metabolism in modern organisms, may also have metabolized HCN on the early Earth. For example, modern nitrogenases—which recent studies suggest are Archean in origin (Stüeken et al., 2015; Parsons et al., 2021)—are known to promiscuously metabolize HCN at their active site (Li et al., 1982; Conradson et al., 1989; Lowe et al., 1989; Pickett et al., 2004; Schwartz et al., 2022). HCN metabolism may have been more prevalent in ancestral versions of these enzyme families.

Variations in age estimates arising from different calibrations or clock models highlights the importance of rigorous testing to identify and mitigate sources of bias and uncertainty in divergence time estimates. For the nitrilases, plant-based calibrations appear to be most consistent in recovering the known ages of other fossil-calibrated clades; animal-based calibrations push the overall age estimates older, while fungi-based calibrations push these estimates younger (Figure 4; Supplementary Table S2). These patterns underscore the benefits of including and evaluating multiple paleontologically-supported calibration schemes in large trees.

Comprehensive gene trees for extant nitrilases, NHases, and SCNases suggest a broad diversity of previously unstudied enzyme variants. As all three enzyme groups have been previously identified to have significant potential in bioremediation and biotechnology, these data may prove useful for identifying new candidates of commercial or environmental interest. Enzyme variants that are similar to those with established biocatalytic or biosynthetic value may be useful in expanding the toolset available for targeted nitrile metabolism. Perhaps more importantly, divergent enzymes or host strains may identify candidates with previously-unexplored biochemical potential; for example, previous studies have reported nitrilases or NHases that are more thermostable, more pH-stable, more efficient, or more readily inducible than previous enzyme candidates (Gong et al., 2012; Kuhn et al., 2012; Pei et al., 2013; Supreetha et al., 2019; Shen et al., 2021).

## Data availability statement

The datasets presented in this study can be found in online repositories. The datasets can be found at https://doi.org/10.6084/m9.figshare.c.5952186.

## Author contributions

SS and GF contributed to study design. SS and LR performed data collection and analysis. LR, JP, and SS developed scripts for data processing. SS and JP performed data visualization. GF provided supervision and guidance in data analysis. SS wrote the manuscript with contributions from all authors. All authors contributed to the article and approved the submitted version.

## Funding

This work was supported by a Simons Foundation Collaboration on the Origins of Life grant (#339603) to GF and a National Defense Science and Engineering Graduate Fellowship to SS.

## Conflict of interest

The authors declare that the research was conducted in the absence of any commercial or financial relationships that could be construed as a potential conflict of interest.

## Publisher’s note

All claims expressed in this article are solely those of the authors and do not necessarily represent those of their affiliated organizations, or those of the publisher, the editors and the reviewers. Any product that may be evaluated in this article, or claim that may be made by its manufacturer, is not guaranteed or endorsed by the publisher.
