# Supplementary material for: A Proterozoic microbial origin of extant cyanide-hydrolyzing enzyme diversity
Source: Front Microbiol. 2023 Mar 30;14:1130310. doi: 10.3389/fmicb.2023.1130310 (PMC10098168; doi:10.3389/fmicb.2023.1130310)
Supplement: Supplementary file 1 [file Table_1.pdf]

Supplementary Table S1. Class 1 Nitrilase ancestor age prior and posterior distributions/mean estimates, by calibration and model subset

MODEL	CALIBRATIONS	PRIOR CLASS 1 LCA AGE RANGE (Ma)	PRIOR CLASS 1 LCA MEAN AGE (Ma)	POSTERIOR CLASS 1 LCA AGE RANGE (Ma)	POSTERIOR MEAN CLASS 1 LCA AGE (Ma)
UGAM	Root cal only	3598-1104	2329	3534-1074	2212
UGAM	Fungi	1182-958	1058	1268-972	1108
UGAM	Plant	2306-1226	1689	2469-1230	1762
UGAM	Animal	3652-2137	2808	3536-2053	2724
UGAM	Plant + Animal	2867-1891	2374	2809-1789	2259
UGAM	Plant + Fungi	1189-979	1079	1313-1033	1164
UGAM	Fungi + Animal	1356-1135	1228	1461-1162	1308
UGAM	All calibrations	1356-1098	1229	1468-1182	1318
LN	Root cal only	3542-1114	2228	3562-1104	2230
LN	Fungi	1930-964	1548	1591-1170	1369
LN	Plant	1608-1036	1296	1568-998	1252
LN	Animal	3753-2568	3252	2759-2481	3190
LN	Plant + Animal	2805-1774	2240	2644-1672	2094
LN	Plant + Fungi	1860-1347	1572	1549-1183	1350
LN	Fungi + Animal	2258-1673	1942	1925-1464	1681
LN	All calibrations	2112-1572	1839	1877-1446	1626
CIR	Root cal only	3581-1075	2223	3532-1077	2216
CIR	Fungal	1136-892	1001	1147-847	982
CIR	Plant	1506-967	1195	1501-937	1172
CIR	Animal	2510-1327	1800	2640-1382	1876
CIR	Plant + Animal	1680-1208	1414	1736-1196	1437
CIR	Plant + Fungi	1139-916	1021	1141-892	1004
CIR	Fungi + Animal	1267-1006	1139	1267-1006	1130
CIR	All calibrations	1257-1026	1143	1251-1018	1125
