# Supplementary material for: A Proterozoic microbial origin of extant cyanide-hydrolyzing enzyme diversity
Source: Front Microbiol. 2023 Mar 30;14:1130310. doi: 10.3389/fmicb.2023.1130310 (PMC10098168; doi:10.3389/fmicb.2023.1130310)
Supplement: Supplementary file 2 [file Table_2.pdf]

Supplementary Table S2. Age estimates for fossil-constrained clades by other calibrations

**Node/ Cal.**	**Constraint age input range (Ma)**	**Age with Osteichthyes calibration (Ma), range/mean Posterior, *prior***	**Age with plant calibration (Ma), range/mean Posterior, *prior***	**Age with fungal calibrations (Ma), range/mean Posterior, *prior***	**Age with Plant+Fungal calibrations (Ma), range/mean Posterior, *prior***	**Age with Plant+ Animal calibrations (Ma), range/mean Posterior, *prior***	**Age with Fungal+ Animal calibrations (Ma), range/mean Posterior, *prior***	**Age with root-only calibrations (Ma), range/mean Posterior, *prior***
Osteichthyes	467-445	--	U: 422-165/272<br>*356-156/ 244*<br>L: 178-68/114<br>*173-66/111*<br>C: 423-192/ 294<br>*430-211/ 310*	U: 235-117/171<br>*220-110/157*<br>L: 199-69/128<br>*220-87/147*<br>C: 329-166/244<br>*330-168/ 243*	U: 245-124/ 178<br>*220-115/164*<br>L: 195-70/120<br>*206-75/133*<br>C: 338-166; 249<br>*337-180; 257*	--	--	U: 594-154/342<br>*592-16/ 350*<br>L: 437-84/212<br>*378-90/210*<br>C: 964-253/ 561<br>949-249/552
Plantae	750-590	U: 1735-707/1145<br>*1792-762/ 1216*<br>L: 2266-1316/1803<br>*2234-1256/1761*<br>C: 1640-796/ 1138<br>*1536-754/ 1093*	--	U: 660-320/469<br>*620-318/461*<br>L: 936-592/760<br>*1059-616/833*<br>C: 713-466/ 590<br>*696-465/ 583*	--	--	U: 746-366/ 546<br>*682-360/510*<br>L: 1110-703/895<br>*1202-762/966*<br>C: 785-523/659<br>*783-519/655*	U: 1658-415/ 934<br>*1732-451; 988*<br>L: 2115-615/<br>*2021-600/ 1233*<br>C: 2195-640/ 1342<br>*2162-620/1310*
Fungi (1)	350-250	U: 946-470/687<br>*1035-528/ 752*<br>L: 746-323/502<br>*606-272/ 423*<br>C: 740-301/479<br>*689-327/483*	U: 648-278/437<br>*634-295/440*<br>L: 293-122/195<br>*266-109/174*<br>C: 434-205/314<br>*480-221/325*	--	--	U: 763-407/567<br>*809-434/ 615*<br>L: 497-199/316<br>*446-179/ 298*<br>C: 570-254/ 376<br>*496-265/ 369*	--	U: 924-257/554<br>*1034-272/614*<br>L: 637-149/345<br>*509-130/284*<br>C: 1332-377/ 805<br>*1242-344/ 740*
Fungi (2)	350-250	U: 1059-494/737<br>*1104-543/ 807*<br>L: 1107-531/782<br>*911-495/695*<br>C: 954-413/638<br>*960-486/673*	U: 696-289/460<br>*696-323/471*<br>L: 400-188/285<br>*372-181/265*<br>C: 552-277/ 398<br>*552-305/424*	--	--	U: 851-405/601<br>*895-470/660*<br>L: 690-326/483<br>*658-305/ 468*<br>C: 674-348/ 506<br>*652-382/ 513*	--	U: 1006-272/584<br>*1070-301/644*<br>L: 966-244/538<br>*825-231/485*<br>C: *1282-359/ 764*<br>*1360-388/822*
Fungi (3)	350-250	U:1150-606/855<br>*1104-543/ 807*<br>L: 1159-614/870<br>*947-548/735*<br>C: 934-460/652<br>*931-456/643*	U: 790-364/550<br>*822-415/593*<br>L: 430-236/322<br>*372-181/265*<br>C: 561-315/ 418<br>*532-318/411*	--	--	U: 1208-663/908<br>*1027-637/ 818*<br>L: 732-382/543<br>*658-305/ 468*<br>C: 725-433/ 535<br>*609-400/ 493*	--	U: 1143-329/695<br>*1291-354; 810*<br>L: 1052-282/606<br>*830-250/510*<br>C: 1291-373/ 783<br>*1302-380; 795*
Fungi (4)	490-400	U: 1482-775/1092<br>*1638-821/ 1172*<br>L: 1479-811/1133<br>*1504-667/ 1074*<br>C: 1338-626/ 910<br>*1280-620/ 904*	U: 1020-472/700<br>*966-482/698*<br>L: 594-307/443<br>*548-316/425*<br>C: 740-353/549<br>*730-448/578*	--	--	U: 936-536/712<br>*1265-731/ 968*<br>L: 898-429/627<br>*838-423; 612*<br>C: 875-520/ 697<br>*830-495/ 651*	--	U: 1450-414/ 884<br>*1554-443/963*<br>L: 1402-379/813<br>*1288-362/762*<br>C: 1816-512/ 1091<br>*1776-487/ 1056*
Fungi (5)	680-410	U: 1562-854/1169<br>*1577-942/ 1225*<br>L: 1481-817; 1105<br>*1212-742/ 958*<br>C: 1154-591/815<br>*1140-587/ 803*	U: 1064-505/747<br>*1015-529/733*<br>L: 531-299/403<br>*481-253/352*<br>C: 691-398 / 524<br>*730-448/578*	--	--	U: 1262-747/ 977<br>*1274-774/ 1019*<br>L: 922-513/690<br>*856-436/ 631*<br>C: 824-524/ 656<br>*748-499/ 612*	--	U: 1531-452/ 942<br>*1560-484/997*<br>L: 1249-357/752<br>*1047-316/650*<br>C: 1598-474/983<br>*1592-476/ 987*

Clock Models: U--UGAM; L--LN; C--CIR clock models

Red: estimated age range does NOT intersect calibration age range for node

Black: estimated age range intersects calibration age range, mean age does not

Blue: estimated node age range and mean age intersect calibration age range
