# Supplementary material for: A Proterozoic microbial origin of extant cyanide-hydrolyzing enzyme diversity
Source: Front Microbiol. 2023 Mar 30;14:1130310. doi: 10.3389/fmicb.2023.1130310 (PMC10098168; doi:10.3389/fmicb.2023.1130310)
Supplement: Supplementary file 3 [file Table_3.pdf]

Supplementary Table S3. Age variance calculated for triplicate fungal clades by clock model

Calibration subset	Prior/Posterior	Variance, UGAM	Variance, LN	Variance, CIR
Animal	Prior	10073.13	19266.38	6959.36
Animal	Posterior	4938.04	24696.60	6125.44
Plant	Prior	4357.46	2038.49	1949.36
Plant	Posterior	2371.88	2861.05	2030.22
Animal+Plant	Prior	7569.54	7141.20	4055.56
Animal+Plant	Posterior	3843.81	9206.00	4797.94
None	Prior	7420.20	10244.72	1183.75
None	Posterior	3681.61	12255.40	288.61

minimized variance in gray
