# Supplementary material for: A Proterozoic microbial origin of extant cyanide-hydrolyzing enzyme diversity
Source: Front Microbiol. 2023 Mar 30;14:1130310. doi: 10.3389/fmicb.2023.1130310 (PMC10098168; doi:10.3389/fmicb.2023.1130310)
Supplement: Supplementary file 4 [file Image_1.pdf]

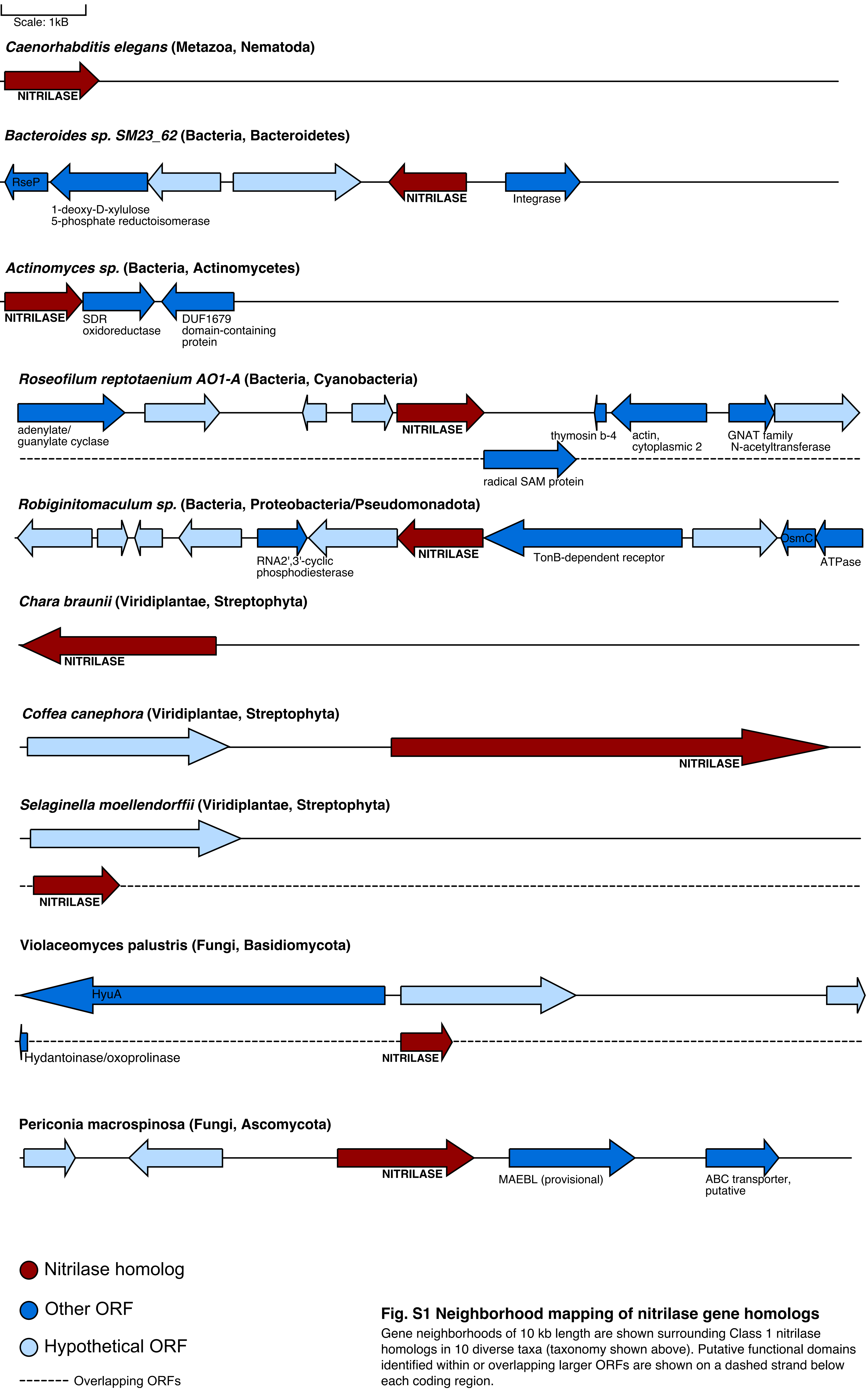

Fig. S1 Neighborhood mapping of nitrilase gene homologs

Gene neighborhoods of 10 kb length are shown surrounding Class 1 nitrilase homologs in 10 diverse taxa (taxonomy shown above). Putative functional domains identified within or overlapping larger ORFs are shown on a dashed strand below each coding region.
