# Supplementary figures and images for: A Proterozoic microbial origin of extant cyanide-hydrolyzing enzyme diversity
Source: Front Microbiol. 2023 Mar 30;14:1130310. doi: 10.3389/fmicb.2023.1130310 (PMC10098168; doi:10.3389/fmicb.2023.1130310)

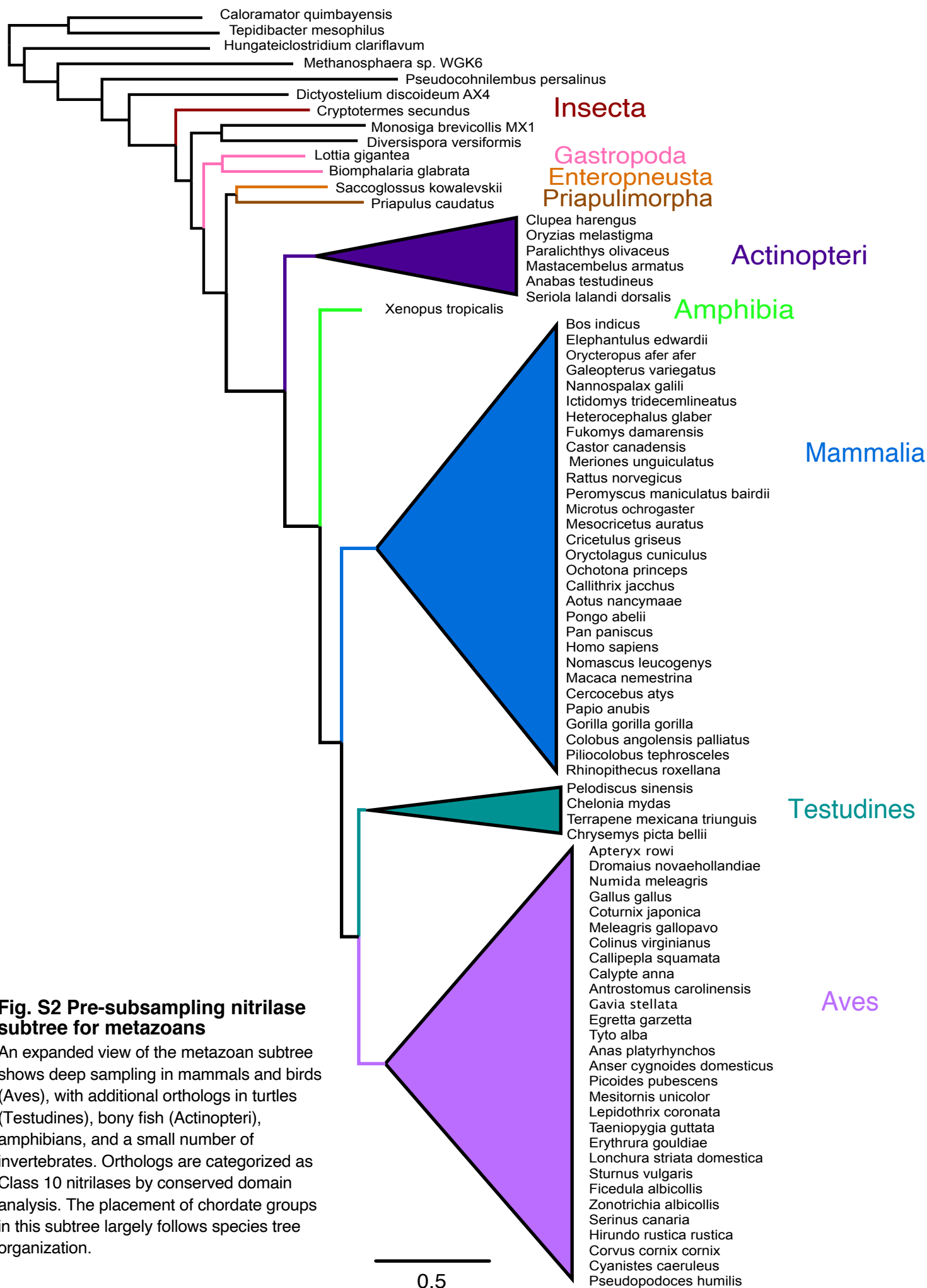

Supplement: Supplementary file 5 [file Image_2.pdf]
