# Supplementary material for: A Proterozoic microbial origin of extant cyanide-hydrolyzing enzyme diversity
Source: Front Microbiol. 2023 Mar 30;14:1130310. doi: 10.3389/fmicb.2023.1130310 (PMC10098168; doi:10.3389/fmicb.2023.1130310)
Supplement: Supplementary file 6 [file Image_3.pdf]

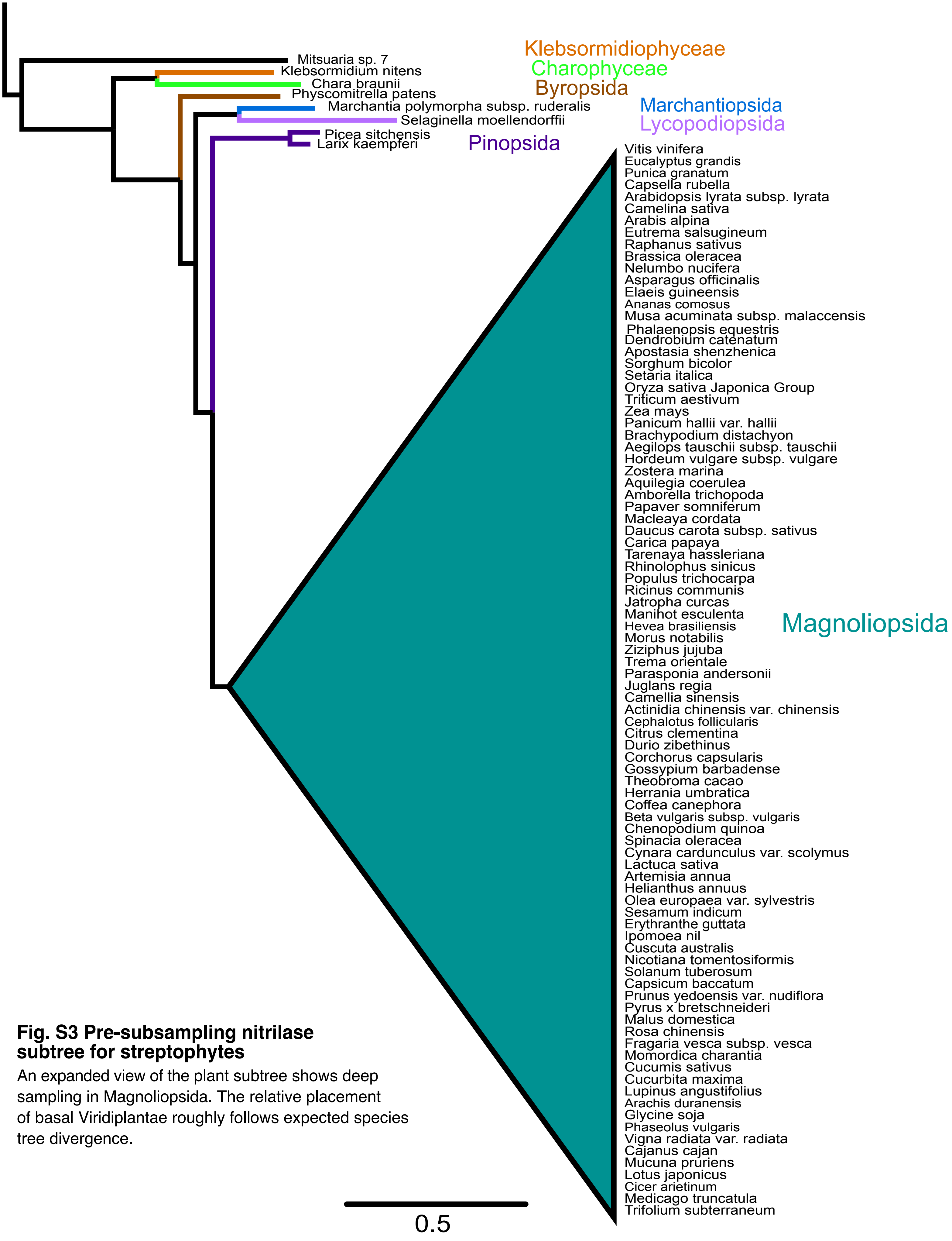

Fig. S3 Pre-subsampling nitrilase subtree for streptophytes

An expanded view of the plant subtree shows deep sampling in Magnoliopsida. The relative placement of basal Viridiplantae roughly follows expected species tree divergence.
